# Comparison of venous plasma glycemia and capillary glycemia for the screening of type 2 diabetes mellitus in the Japanese-Brazilian community of Mombuca (Guatapará-SP)

**DOI:** 10.1186/1758-5996-2-6

**Published:** 2010-01-22

**Authors:** Maria Cristina Foss-Freitas, Regina CG de Andrade, Roberta C Figueiredo, Ana Emília Pace, Edson Z Martinez, Amaury L Dal Fabro, Laércio J Franco, Milton C Foss

**Affiliations:** 1 Departamento de Clinica Médica, Faculdade de Medicina de Ribeirão Preto-USP, (Av. Bandeirantes 3900), Ribeirão Preto-SP(14049-900), Brazil; 2 Faculdade de Ciências Farmacêuticas de Ribeirão Preto-USP, (Av. Bandeirantes 3900), Ribeirão Preto-SP(14049-900), Brazil; 3 Departamento de Medicina Social, Faculdade de Medicina de Ribeirão Preto-USP, (Av. Bandeirantes 3900), Ribeirão Preto-SP(14049-900), Brazil; 4 Escola de Enfermagem de Ribeirão Preto-USP, (Av. Bandeirantes 3900), Ribeirão Preto - SP (14040-902), Brazil

## Abstract

**Background:**

To identify the most appropriate cut-off points of fasting glycemia for the screening of diabetes mellitus type 2 (DM2) with the comparison of the properties of capillary glycemia (CG) and venous blood plasma glycemia (PG) in a population of Japanese origin from the community of Mombuca, Guatapará - SP, Brazil.

**Methods:**

This was a population-based descriptive cross-sectional study conducted on a sample of 131 individuals of both genders aged 20 years or more (66.8% of the target population). CG was measured with a glucometer in a blood sample obtained from the fingertip and PG was determined by an enzymatic method (hexokinase) in venous blood plasma, after a 10-14 hour fast in both cases. Data were analyzed by the receiver operating characteristic (ROC) curve in order to identify the best cut-off point for fasting glycemia (CG and PG) for the diagnosis of DM, using the 2-hour plasma glycemia > 200 mg/dl as gold - standard.

**Results:**

The ROC curve revealed that the best cut-off point for the screening of DM was 110 mg/dl for CG and 105 mg/dl for PG, values that would optimize the relation between individuals with positive and false-positive results. The area under the ROC curve was 0.814 for CG (p < 0.01) and 0.836 for PG (p < 0.01).

**Conclusions:**

The cut-off points of 105 mg/dl(5.8 mmol/l) for PG and of 110 mg/dl(6.1 mmol/l) for CG appear to be the most appropriate for the screening of DM2 in the population under study, with emphasis on the fact that the value recommended for CG is 5 mg/dl higher than that for PG, in contrast to WHO recommendations.

## Background

Diabetes mellitus type 2 (DM2) is a highly prevalent disease of epidemic projection for the next few years both in developed and developing countries [1], representing an important public health problem in view of the high morbidity and mortality of the disease itself and the high costs involved in its control and in the treatment of its complications. DM2 is among the 10 major causes of death in several industrialized and emergent countries [2,3]. In 2002, the cost of treating diabetic patients in the US was more than double the cost of treating non-diabetic individuals, amounting to approximately US$132 billion [4].

Over the last few years, a worldwide trend has been observed showing that migrant populations present a pattern of morbidity-mortality that differs from that of the community of origin of their parents, but follows the disease profile of local residents. Epidemiological studies have demonstrated changes in the prevalence of non-transmissible chronic diseases, DM2 in particular, among Japanese migrants and their descendants. In this respect, higher prevalences of DM2 (16-20%) have been observed among Japanese residing in the US than among Japanese residing in Japan (4-5%) [5,6]. Similarly, a high prevalence of DM2 [7,8] (36.2%) and of impaired glucose tolerance (IGT = 23.4%) have been detected among Japanese individuals living in Brazil, corresponding to higher values than those detected in the Brazilian population [9]. These changes are explained by modifications of life style, with the incorporation of new cultural patterns and changes in eating habits. However, other factors also participate in this process, such as greater population longevity, miscegenation and the sociodemographic transformation due to urbanization and economic development [10].

Despite the proven benefit of early detection of the disease [11], universal screening for DM2 has been questioned [12]. The most accepted recommendation is the screening of asymptomatic individuals with a greater risk for the disease [13-15], using the determination of fasting plasma glucose level as the worldwide accepted method for screening and diagnosis. On the other hand, in view of its practical application, agility, rapidity and safety, the capillary glycemia (CG) test is an important option for the screening of DM in detection campaigns or in population studies [16].

The objective of the present population-based study was to identify the best cut-off points of fasting glycemia for the screening of DM2 and to compare the properties of fasting capillary glycemia (CG) and fasting venous plasma glycemia (PG) by the analysis of the areas under the receiver operating characteristic (ROC) curve [17] in a population of Japanese origin living in the community of Mombuca, Guatapará - SP, Brazil.

## Methods

The data were obtained in a cross-sectional descriptive study conducted on the Japanese-Brazilian population of Mombuca, Guatapará - SP, during the period from April to December 2005. Among 1^st ^and 2^nd ^generation individuals (Issei and Nissei, respectively) of both genders and older than 20 years, 131 (66.8% of the population in this age range) agreed to participate in the study. The study was approved by the Research Ethics Committee of the Health Teaching Center of the Faculty of Medicine of Ribeirão Preto-USP, under protocol n° 104/03 and all subjects gave written informed consent to participate.

On a scheduled date, each participant came to the office of the Agricultural and Sports Association of Guatapará for information about the study. After a fasting period of 10 to 14 hours, a venous blood sample was obtained from each subject for PG determination and a blood sample was obtained from a fingertip for the determination of CG. The blood samples were collected into vacuum tubes containing sodium fluoride and used for PG determination by enzymatic method (hexokinase) with a Cobas Mira Plus Analyser. CG was measured with a glucometer (Advantage-Roche) and reported as whole blood glucose level. Individuals presenting CG of less than 200 mg/dl were then asked to perform the 75 g glucose tolerance test, excluding those with previously diagnosed diabetes.

Subjects were considered to have diabetes mellitus when their fasting glycemia was ≥ 126 mg/dl(7.0 mmol/l) or when their glycemia was ≥ 200 mg/dl(11.1 mmol/l) 2 hours after 75 g glucose load, or if they were under treatment for diabetes. Subjects were considered to be pre-diabetic when their fasting glycemia was 110(6.1 mmol/l) to 126 mg/dl (impaired fasting glycemia) and/or when their glycemia was 140(7.8 mmol/l) to 200 mg/dl 2 hours after glucose overload (impaired glucose tolerance). This diagnostic criteria for diabetes or pre-diabetes was based on plasma glucose levels [16].

### Statistical Analysis

CG and PG data were compared between the diabetic and non-diabetic groups by the Student *t-*test for independent samples, with the level of significance set at p < 0.05. CG and PG were compared in terms of sensitivity and specificity using the ROC curve, considering the diagnostic standard for diabetes mellitus to be PG ≥ 200 mg/dl(11.1 mmol/l) 2 hours after the ingestion of 75 g anhydrous glucose. The criterion used to select the cut-off points of the fasting glycemias (CG and PG) was based on the values with the closest sensitivity and specificity. The areas under the ROC curve for fasting PG and CG were compared by the nonparametric test proposed by DeLong et al. [18]. For interpretation of the results, it is considered that the greater the area under the ROC curve, the greater the discriminant power of the test for a determined outcome.

## Results

The clinical data of the participants are presented on Table 1. Among the 131 individuals evaluated, five were excluded because they were previously diagnosed diabetics and nine did not perform the CG test. Among the subjects without DM, mean CG was significantly higher (8%) than PG, whereas among subjects with DM the differences were nonsignificant, although mean CG was 5% higher than PG (Table 2). The prevalence of DM and IGT in this group were 13.7% and 14.5%, respectively, and the frequency of hypertension (>140/90 mmHg) was 48.1%.

**Table 1:** Clinical data of the Japanese-Brazilian population of Mombuca, Guatapará-SP, 2005 (Mean ± SD).

	Total *(N = 131)*	Men *(N = 54*)	Women *(N = 77*)
Age (years)	56.7 ± 15.9	55.1 ± 15.9	57.8 ± 16.1
Body Weight *(Kg)*	61.7 ± 14.3	70.6 ± 14.5	55.2 ± 10.2
Height *(m)*	1.6 ± 0.1	1.7 ± 0.1	1.5 ± 0.1
BMI *(kg/m^2^)*	24.7 ± 4.0	25.4 ± 4.2	24.1 ± 3.9
Waist Circumference *(cm)*	84.9 ± 10.6	88.6 ± 10.1	83.1 ± 10.7

**Table 2:** Fasting CG and PG levels of the Japanese-Brazilian population of Mombuca, Guatapará-SP, 2005 (Mean ± SD).

GLYCEMIA	Normoglycemic + IGT	DM	p-value*
CG (mg/dl) (N = 117)	95.9 ± 15.8	120.5 ± 19.1	< 0.0001
PG (mg/dl) (N = 126)	88.4 ± 8.9	113.8 ± 20.1	< 0.0001
p-value*	< 0.0001	0.4140	

* Student t-test

**Figure 1:**
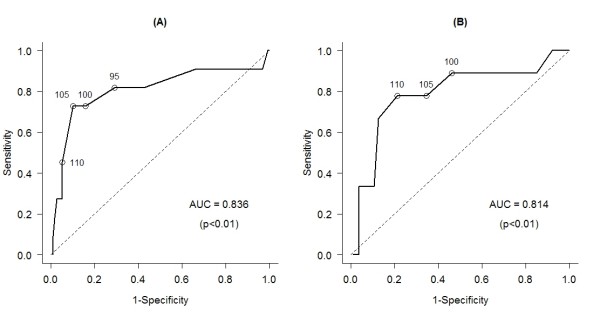
**ROC curve for fasting PG (A) and CG (B) for the identification of DM in the Japanese-Brazilian population of Mombuca, Guatapará-SP, 2005**.

The ROC curve revealed that the ideal cut-off point for DM screening was 105 mg/dl for PG and 110 mg/dl for CG (Fig. 1), values that would optimize the relation between subjects with positive and false-positive results. The level of 110 mg/dL in CG showed 77.8% sensitivity and 82.% specificity and the level of 105 mg/dL in PG showed 72.7% sensitivity and 93.0% specificity. It can be seen that PG showed a better power for the detection of DM, with a greater area under the ROC curve (0.836 - p < 0.01) compared to CG (0.814 - p < 0.01), although the difference was nonsignificant (p = 0.66).

## Discussion

The high prevalence of DM and the epidemic projections of the disease for the next few years in both developed and developing countries are a source of concern for the health area authorities. In addition, the fact that 35 to 50% of the individuals with DM do not know that they have the disease and that at the time of diagnosis the patients already present micro- and macrovascular complications, supports the importance of establishing an early diagnosis of DM, which would prevent the occurrence of many of these complications [19,20].

The comparison of glycemia values obtained from capillary blood and from venous plasma blood is a subject of extensive discussion [21-25]. However, it is known that the level of glucose in blood after a few hours of fasting varies according to the method employed for determination and the material used, i.e., capillary blood, venous blood, and serum/plasma or whole blood [26].

In a study on the validation of CG versus PG for the detection of DM and IGT, Kruijshoop et al. [22] detected a strong correlation between the two parameters both in the fasting condition (r = 0.92) and in the postprandial condition (r = 0.82), thus demonstrating the validity of the use of CG in the screening phase of epidemiological studies. In agreement with our results, the CG values detected by Kruijshoop et al. [22] were higher than PG values both for diabetic and normoglycemic or IGT subjects in the fasting condition.

Measurements made in plasma yield a 14 to 15% higher result compared to methods that use whole venous blood, which in turn yield about 5% lower glycemia results compared to capillary blood [14,26]. However, according to the WHO [16], under fasting conditions, plasma venous glycemia values are about 10% higher than CG values.

Analysis of the sensitivity and specificity of diagnostic tests by constructing the ROC curve has been recommended in epidemiological studies. In the present study, the cut-off points with the best specificity and sensitivity for CG and PG obtained on the basis of the ROC curve were 110 mg/dl and 105 mg/dl, respectively. The areas under the curve for CG and PG were quite close, 0.814 and 0.836, respectively, showing the expressive capacity of the tests in detecting DM. However, when the areas are superimposed, a separation of the 105 and 110 mg/dl points is noted, suggesting that, even though the literature shows that PG values are higher than CG values, regarding the population studied here, PG values of 105 mg/dl and CG values of 110 mg/dl should be used.

It is important to point out a limitation of this study for the proposed objectives by the number of participants, but the use of CG showed useful for decision taking immediately, i.e., to perform or not a 75 g glucose OGTT.

## Conclusions

In conclusion, our data indicate that the cut-off points of 105 mg/dl(5.8 mmol/l) and 110 mg/dl(6.1 mmol/l) for PG and CG, respectively, are highly sensitive and specific for the diagnosis of DM2 and therefore would be appropriate for the screening of DM2 in this study population. It is important to give emphasis to the fact that the value recommended for CG is 5 mg/dl higher than that for PG, in contrast to WHO recommendations.

## Competing interests

The authors declare that they have no competing interests.

## Authors' contributions

MCFF, LJF and MCF participated in the design of the study. MCFF, RCGA, RCF, AEP, ALDF, LJF and MCF performed the data collection. EM performed the statistical analysis. MCFF, RCGA, LJF and MCF wrote the paper. All authors read and approved the final manuscript.

## References

[B1] King H Aubert RE Herman WH Global burden of diabetes, 1995-2025: prevalence, numerical estimates, and projections Diabetes Care 1998 21 1414 1431 10.2337/diacare.21.9.1414 9727886

[B2] Wild S Roglicroglic G Green A Sicree R King H Global prevalence of diabetes. Estimates for the year 2000 and projections for 2030 Diabetes Care 2004 27 1047 1053 10.2337/diacare.27.5.1047 15111519

[B3] Brasil. Ministério da Saúde. Secretaria de Atenção à Saúde. Departamento de Atenção Básica Diabetes Mellitus/Cadernos de Atenção Básica-no.16, Normas e Manuais Técnicos, Brasília-DF 2006 64

[B4] American Diabetes Association Economic costs of diabetes in the U.S. in 2002 Diabetes Care 2003 26 917 932 10.2337/diacare.26.3.917 12610059

[B5] Fujimoto WY Leonetti DL Kinyoun JL Newell-Morris LL Shuman WP Stolov WC Whal PW Prevalence of diabetes mellitus and impaired glucose tolerance among second generation Japanese American men Diabetes 1987 36 721 729 10.2337/diabetes.36.6.721 3569671

[B6] Hara H Egusa G Yamakido M Kawate R The high prevalence of diabetes mellitus and hyperinsulinemia among the Japanese-Americans living in Hawaii and Los Angeles Diabetes Res Clin Pract 1994 24 Suppl S37 S42 10.1016/0168-8227(94)90225-9 7859631

[B7] Franco LJ Gimeno SGA Ferreira SRG Iunes M Incremento na Mortalidade associada à presença de Diabetes Mellitus em nipo-brasileiros Revista de Saúde Pública 1998 32 118 124 10.1590/s0034-89101998000200003 9713115

[B8] Gimeno SGA Ferreira SRG Franco LJ Hirai AT Matsumura RS Moisés RS Prevalence and 7-year incidence of Type II diabetes mellitus in a Japanese-Brazilian population: an alarming public health problem Diabetologia 2002 45 1635 1638 10.1007/s00125-002-0963-x 12488952

[B9] Malerbi DA Franco LJ Multicenter study of the prevalence of diabetes mellitus and impaired glucose tolerance in urban Brazilian population aged 30-69 years Diabetes Care 1992 15 1509 1516 10.2337/diacare.15.11.1509 1468278

[B10] Grupo de Estudos de Diabetes na Comunidade Nipo-Brasileira (JBDSG) Diabetes Mellitus e Doenças Associadas em Nipo-Brasileiros 2004 São Paulo: Gree Forest do Brasil Editora

[B11] UK Prospective Diabetes Study(UKPDS)Group Intensive blood-glucose control with sulphonylureas or insulin compared with conventional treatment and risk of complications in patients with type 2 diabetes (UKPDS 33) Lancet 1998 352 837 853 10.1016/S0140-6736(98)07019-6 9742976

[B12] Georg AE Duncan BB Toscano CM Schmidt MI Mengue S Duarte C Polaczyk CA Economic analysis of a screening program for diabetes mellitus in Brazil Rev Saúde Pública 2005 39 452 460 10.1590/S0034-89102005000300017 15997322

[B13] American Diabetes Association Standards of Medical Care in Diabetes Diabetes Care 2008 31 s1 S5 S11

[B14] American Diabetes Association Screening for Type 2 Diabetes Diabetes Care 2003 26 s1 S21 S24 12502615 10.2337/diacare.26.2007.s21

[B15] Oliveira JEP Milech A Diabetes Mellitus- Clínica, Diagnóstico e Tratamento Multidisciplinar 2004 São Paulo: Editora Atheneu

[B16] Worldworld Health Organization Definition, diagnosis and classification of diabetes mellitus and its complications. Report of WHO Consultation. Part 1: Diagnosis and Classification of Diabetes Mellitus 1999 Genebra WHO

[B17] Pagano M Gauvreau K Princípios de Bioestatística 2006 São Paulo: Thomson Learning

[B18] Delong ER Delong DM Clarke-Pearson DL Comparing the areas under two or more correlated receiver operating characteristic curves: a non parametric approach Biometrics 1988 44 837 845 10.2307/2531595 3203132

[B19] Harris MI Eastman RC Early detection of undiagnosed diabetes mellitus: a US perspective Diabetes Metab Res Rev 2000 16 230 236 10.1002/1520-7560(2000)9999:9999<::AID-DMRR122>3.0.CO;2-W 10934451

[B20] Engelgau MM Narayan KMV Herman WH Screening for type 2 diabetes Diabetes Care 2000 23 1563 1580 10.2337/diacare.23.10.1563 11023153

[B21] Colagiuri S Sandbaek A Carstensen B Christensen J Glumer C Lauritzen T Borch-Jonhsen K Comparability of venous and capillary glucose measurements in blood Diabetic Medicine 2003 20 953 956 10.1046/j.1464-5491.2003.01048.x 14632723

[B22] Kruijshoop M Feskens EJM Blaak EE Bruin TWA Validation of capillary glucose measurements to detect glucose intolerance or type 2 diabetes mellitus in the general population Clinica Chimica Acta 2004 341 33 40 10.1016/j.cccn.2003.10.033 14967156

[B23] Kuwa K Nakayama T Hoshino T Tominaga M Relationships of glucose concentrations in capillary whole blood, venous whole blood and venous plasma Clinica Chimica Acta 2001 307 187 192 10.1016/S0009-8981(01)00426-0 11369356

[B24] Stahl M Brandslund I Jorgensen LG Hyltoft Petersen P Borch-Johnsen K de Fine Olivarius N Can capillary whole blood glucose and venous plasma glucose measurements be used interchangeably in diagnosis of diabetes mellitus? Scand J Clin Lab Invest 2002 62 159 166 10.1080/003655102753611799 12004932

[B25] Stahl M Brandslund I Measurement of glucose content in plasma from capillary blood in diagnosis of diabetes mellitus Scand J Clin Lab Invest 2003 63 431 440 10.1080/00365510310002590 14594324

[B26] Arduino F Arduino F Sintomas, Diagnóstico, Prognóstico e Mortalidade do Diabetes Diabetes Mellitus 1980 3 Rio de Janeiro: Editora Guanabara Koogan 78 94

